# Puzzle-based pedagogic activity to address rote learning of antibiotic classes in clinical pharmacology course for undergraduate nursing students

**DOI:** 10.1186/s12909-026-09484-1

**Published:** 2026-05-28

**Authors:** Constanza Morén, Emi Ruiz Mariscal, Mónica Negredo, María Preciado, Gemma Martínez, Mireia Llaurado-Serra, Antonio Rosa, Melinda González, Natàlia Rodríguez, Laura Martínez

**Affiliations:** 1 Clinical and Fundamental Nursing Department, Nursing Faculty, University of Barcelona, Barcelona, Spain; 2 Centro de Investigaciones Biomédicas en Red de Salud Mental, CIBERSAM, Madrid, Spain; 3 Institut d’Investigacions Biomèdiques August Pi i Sunyer, IDIBAPS, Barcelona, Spain; 4 Barcelona Clínic Schizophrenia Unit, BCSU, Barcelona, Spain; 5 Institut Català de la Salut (Catalan Health Institute), Generalitat de Catalunya, Barcelona, Spain; 6 Department of Laboratories and Pharmacy, Institut Hospital del Mar de Formació Professional Sanitària, Barcelona, Spain; 7 Deputy of Strategy and Planning Direction, Hospital Clínic i Provincial de Barcelona, Barcelona, Spain; 8 Basic and Clinical Practice Department, Faculty of Medicine and Health Sciences, University of Barcelona, Barcelona, Spain; 9 Nursing Faculty, Campus Bellvitge, Feixa Llarga, s/n, l’Hospitalet de Llobregat, 08907 Spain; 10 Faculty of Medicine and Health Sciences, C/Casanova, 143, Barcelona, 08036 Spain

**Keywords:** Antibiotics, Clinical Pharmacology, Nursing students, Puzzle activity

## Abstract

**Objective:**

We aimed to evaluate the impact of an active learning strategy, the Puzzle technique, on nursing students’ knowledge acquisition and engagement in Clinical Pharmacology, focusing on antibiotics.

**Background:**

Absenteeism and low motivation among university students pose significant challenges in higher education. Traditional rote learning methods often lead to disengagement, particularly in content-heavy subjects such as Clinical Pharmacology. Within this field, learning antibiotic classes requires extensive memorization, which may hinder students’ performance and retention of knowledge. Given the increasing role of nurses in antibiotic prescription, enhancing their pharmacological training is essential.

**Design:**

Educational intervention study with three intervention groups and two control groups, including within-group pre-post assessments.

**Methods:**

A total of 330 s-year nursing students from the University of Barcelona participated. Three groups (A, B, and C) engaged in the Puzzle intervention, while two groups (D and E) served as controls, thereby receiving only the standard theoretical instruction without any additional activity. The intervention consisted of a peer-to-peer collaborative activity where students were assigned specific antibiotic topics to teach their peers. Learning outcomes were assessed through a quiz, midterm, and final exams.

**Results:**

Intervention groups demonstrated significantly higher accuracy in quiz (*p* < 0.001) and exam accuracy percentages (*p* < 0.001) compared to controls. Among the intervention groups, students who participated in the activity outperformed their intra-group controls. Additionally, students reported high satisfaction and perceived applicability in learning consolidation.

**Conclusions:**

Our findings specifically pertain to undergraduate nursing students and undergraduate nursing education. The Puzzle technique effectively enhanced knowledge retention of antibiotic drug classes within Clinical Pharmacology. This peer-to-peer approach may serve as a valuable pedagogical tool for improving learning outcomes in nursing education, particularly in complex pharmacological subjects.

**Supplementary Information:**

The online version contains supplementary material available at 10.1186/s12909-026-09484-1.

## Introduction

The World Health Organization has identified antimicrobial resistance as a critical global health threat [1], necessitating urgent action to promote the rational use of antibiotics and to implement effective management and supervision programs, such as antimicrobial stewardship [2]. In Spain, registered nurses have recently expanded their competencies to prescribe certain medications, including antibiotics for uncomplicated urinary tract infections in women over 14 years of age. This measure, published in the Official State Gazette recently [3], aims to improve efficiency in primary care and reduce the burden on emergency services. All these realities must be accompanied by adequate training of healthcare personnel. Thus, it is imperative that future nursing professionals are well-prepared to face the clinical reality, which is becoming increasingly complex.

Absenteeism and lack of motivation among university students are increasing trends, posing significant challenges in higher education [4, 5]. Traditional rote learning approaches are often poorly received, as they demand a level of cognitive engagement that many students find difficult to sustain [6]. Clinical Pharmacology is often perceived as a particularly complex subject by students. Recent studies report that a substantial proportion of learners feel overwhelmed by the large volume of content and struggle to apply pharmacological knowledge in practice [7]. Moreover, pharmacology has consistently been described as an inherently difficult discipline across different health professions, especially regarding the transfer of theoretical knowledge into clinical contexts [8]. These findings reinforce the need to explore interactive and learner-centered approaches, to enhance motivation and consolidate learning. In our experience teaching nursing students, we have observed a particular aversion towards a specific section of the curriculum that is both technically dense and requires significant memorization. This observation aligns with studies suggesting that content-heavy courses often lead to disengagement and reduced academic performance [9, 10]. The assessment of nursing students’ competence is a matter of concern worldwide and the complexity of assessing students’ clinical competence has challenged educators for decades [11].

This challenging section within the framework of Clinical Pharmacology focuses on antibiotics, where students are expected to familiarize themselves with various antibiotic classes, their mechanisms of action, clinical indications, contraindications, and associated adverse drug reactions (ADRs). The complexity of this topic lies in the extensive content, which includes a vast array of nomenclatures, mechanisms, and clinical considerations [12], as considered in the teaching plan of the Clinical Pharmacology course within the context of the second year of the Nursing degree program at the University of Barcelona (UB).

Based on our experience, despite this systematic approach, students consistently report difficulty mastering the material due to its reliance on memorization, compounded by the breadth and complexity of the content [13]. Such negative perceptions could be followed by suboptimal learning on this relevant matter and, eventually, suboptimal performance in the clinical setting once the students become graduated clinical nurses working at the health system [14].

To address this pedagogical challenge, we implemented an interactive enhancement tool for teaching support, the Jigsaw classroom, from now on, Puzzle technique, as a means to promote peer-to-peer learning and reduce the cognitive burden of memorization, in the second-year Clinical Pharmacology course of the nursing degree program at UB. The Puzzle technique consists of collaborative learning method developed to foster active learning and deeper comprehension, encouraging students to work collaboratively in groups where each member takes responsibility for a piece of the content [15, 16]. By promoting shared responsibility and peer-to-peer learning, this method aims to enhance student engagement and reduce the cognitive burden of memorization. Recent evidence supports the effectiveness of the Puzzle technique in nursing education. A 2024 systematic review and meta-analysis synthesizing 11 studies from six countries and over 1600 students found that the Puzzle method significantly improves academic achievement, clinical skills, and student attitudes compared to traditional teaching approaches [17]. Positive outcomes included enhanced critical thinking, communication, collaboration, and learner motivation. However, while the technique has been applied across a range of nursing topics, no previous studies have focused specifically on its use in teaching antibiotic pharmacology. This gap highlights the relevance and novelty of the present study, which evaluates the impact of a Puzzle-based intervention in a real-world pharmacology course focused on antibiotics. This study evaluates the effectiveness of this pedagogical approach in addressing the challenges associated with teaching the antibiotic module.

## Methods

### Study setting and context

The activity was implemented across five student groups enrolled at two university campuses (Clínic and Bellvitge). The student groups were pre-existing academic cohorts organized according to the university’s official teaching structure. Group assignment was therefore not randomized. Although no formal matching procedure was conducted, the cohorts followed the same curriculum and academic progression, and were subject to the same assessment criteria throughout the degree program. The term ‘virtual campus’ refers exclusively to the institutional online learning platform used to share supporting materials (e.g., slides, notes, and documents), while all teaching sessions related to the Puzzle activity were conducted in person.

Antibiotics were selected as content based on prior teaching experience, student feedback from previous cohorts, and examination performance, which consistently identified this topic as particularly difficult. To organize the challenging content according to the different existing antibiotic classes [18], the curriculum was structured into five major blocks, based on the volume of information and the instructional materials provided through the virtual campus. The resulting categories were: those disrupting bacterial cell wall or cytoplasmatic membrane synthesis, including (1) penicillins; (2) cephalosporins, carbapenems, and monobactams; and (3) glycopeptides and lipopeptides; (4) those targeting 50 S and 30 S ribosomal subunits, including polymyxins, and daptomycin; and (5) agents targeting DNA, RNA, and folic acid pathways.

### Participants and intervention design

All participating students were enrolled in the second-year Clinical Pharmacology course during the first semester of the academic year in the Nursing Faculty at the UB. All sessions, including the Puzzle activity and regular instruction, were conducted in person, although supporting materials (e.g., lecture notes) were made available through the university’s virtual learning platform. All participants were distributed among 5 groups (A, B, C, D and E). Groups A, B, and C corresponded to three pre-existing teaching cohorts in which the Puzzle activity was offered. Within each of these groups, students who participated in the Puzzle activity constituted the intervention subgroup, whereas students who did not participate served as intra-group controls. Groups D and E were independent teaching cohorts that received standard theoretical instruction only and were therefore considered external control groups. The only difference between B and C or between D and E was logistical, not methodological: both followed the same protocol, but the activity was scheduled on different days and times due to constraints in classroom availability and faculty schedules. In groups A, B and C, the participation in the Puzzle activity was entirely voluntary, and non-participants from these intervention groups were considered intra-group controls. Intra-group comparisons (participants vs. non-participants within the same intervention group) were included to control for potential variability in instructional context and assessment content, allowing for a more accurate estimation of the Puzzle activity’s effect. The two control groups (D and E) belonged to the same academic cohort but were enrolled in different teaching groups at separate university campuses. Both followed the same Clinical Pharmacology curriculum and received standard theoretical instruction without the Puzzle intervention, but completed the evaluative assessments for complementary comparative purposes. Although no individual demographic data (e.g., age or sex) were collected, all participants were second-year undergraduate nursing students enrolled in the same degree program, following an identical academic progression. Based on institutional records, this population is typically composed of students aged between 19 and 22 years, with a predominance of female students (approximately 85–90%), and no relevant demographic differences are expected across groups. A schematic overview of group allocation and comparison strategy is provided in Supplementary Fig. 1.

After the final lecture on antibiotics, the Puzzle activity was implemented across Groups A, B, and C. The activity was designed as a pilot project separated from the curricula. Group A completed the activity during a regular lecture session according to the academic calendar. In contrast, Groups B and C conducted the activity in a designated ad hoc session scheduled outside the standard calendar. This was due to logistical factors, including classroom availability, faculty timetables, and scheduling constraints. These differences were not intentional and occurred both within and across campuses. Although logistical factors such as class schedules and campus organization influenced group allocation, the structure and content of the Puzzle activity were standardized across all intervention groups.

### Activity dynamics

While the classic Puzzle model includes a preliminary expert-group phase, in our adapted version, students were encouraged to prepare their assigned content autonomously. Although they could consult faculty for guidance, if needed, no requests for additional information or orientation were made. They were free to collaborate with peers studying the same topic prior to the group session, but no formal expert meeting was scheduled. This approach was chosen to respect students’ time availability and promote self-directed learning.

**Fig. 1:**
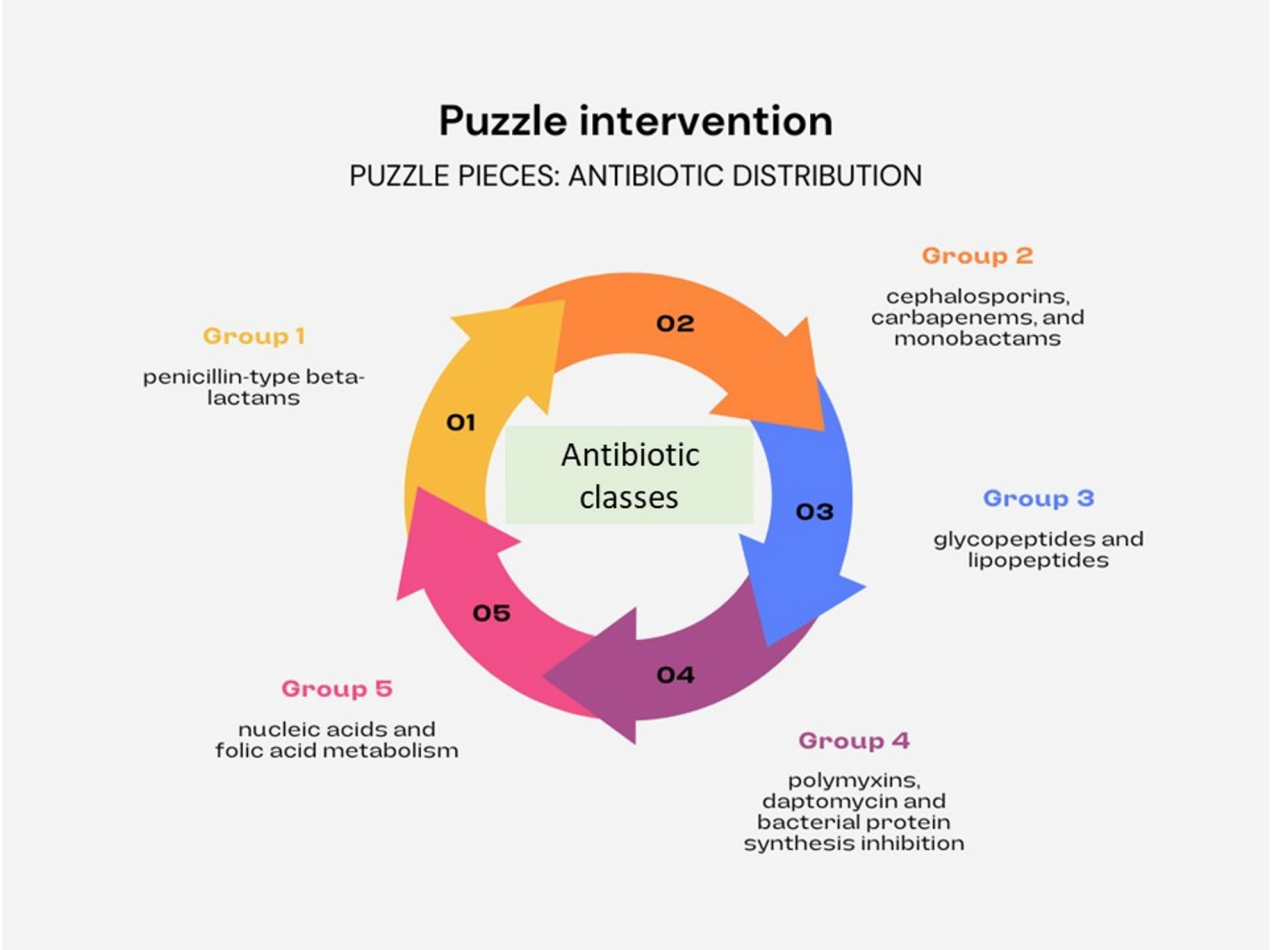
Representative diagram with the distribution of the different groups of antibiotic classes

The activity was structured as follows: each participating student from the intervention groups (A, B and C) was randomly assigned a number from 1 to 5, each corresponding to a specific part of the antibiotic program, and given approximately one week for independent preparation. During the in-person session, students were then randomly reorganized into groups of five, ensuring that each group included one student from each topic block, allowing for comprehensive peer-to-peer teaching. Specifically, numbers 1 and 2 prepared content on beta-lactam antibiotics (those with number 1 covered penicillin-type beta-lactams, and those with number 2 worked on other beta-lactams: cephalosporins, carbapenems and monobactams); number 3 focused on antibiotics targeting the bacterial cell wall, including lipopeptides (e.g., fosfomycin, cycloserine, and bacitracin), and glycopeptides (e.g., vancomycin); number 4 studied inhibitors of bacterial protein synthesis (streptogramins, chloramphenicol, lincosamides, linezolid, macrolides, tetracyclines and aminoglycosides) and agents affecting the bacterial cell membrane (e.g., polymyxins, daptomycin); and number 5 explored antibiotics targeting nucleic acids (e.g., quinolones, metronidazole, nitrofurantoin for DNA, and rifampin for RNA) and folic acid metabolic pathways (e.g., sulfonamides/trimethoprim) (Fig. 1).

The design of the Puzzle activity, during the in-person session lasted between 60 and 90 min, and consisted of forming groups of five, with each member contributing their piece of knowledge to the other four teammates. Taking turns, each participant had 5 to 7 min to present to their group the section of acquired knowledge in each case. Students were free to select their own supporting materials for the activity, ensuring no influence from instructors on the resources used. Students used their own notes and materials they developed (e.g., printed documents, infographics), with lecture slides available through the virtual campus as supplementary resources. Content prepared by students was reviewed and validated by faculty during the sessions to ensure accuracy.

### Evaluation tools for the intervention

The lecture content and the materials for evaluation used in the Puzzle activity were prepared by the same teaching team, ensuring that both approaches covered the same theoretical content on antibiotics. The Puzzle activity was implemented as a pilot educational innovation outside the official curriculum structure; however, the selected topic (antibiotics) was part of the mandatory course content, and therefore its contents were also assessed in the regular quizzes and exams. To assess the potential impact of this peer-to-peer Puzzle activity on learning outcomes, an 11-question quiz was administered to all groups, both intervention and control, for comparative purposes. Although no formal reliability analysis (e.g., Cronbach’s alpha) was conducted, the quiz and exam questions were collaboratively developed and reviewed by experienced Clinical Pharmacology instructors to ensure alignment with course objectives and content validity. The quiz questions are included as supplementary material (Supplementary Material S1). The questionnaire was developed specifically for this study and has not been previously published elsewhere. Additionally, students’ performance on midterm and final exams was analyzed, focusing on questions related to the antibiotic’s content. The quiz and exam questions were collaboratively developed by the course’s teaching team, composed of several faculty members with expertise in Clinical Pharmacology. The materials were reviewed and refined through a consensus process to ensure content validity, alignment with course objectives, and appropriateness for nursing students. Correct and incorrect responses were registered for all groups, regardless of intervention status. In the intervention groups (A, B and C) the responses of the students not participating in the activity were also registered and considered as intra-group controls. Although we have refrained from presenting the complete set of questions from all the exams across all groups in order to maintain the confidentiality of the evaluation tests, a subset of examples from both control and intervened groups is provided (Supplementary Material S2), with the aim of allowing readers to see the type of questions posed. This decision stems from the fact that, although the specific questions vary in each exam, the course outline is limited, and a certain degree of similarity between some questions cannot be entirely avoided.

Both the midterm and final exams were cumulative, covering multiple topics from the Clinical Pharmacology course, including but not limited to antibiotics; however, for this study only the questions related to antibiotics were considered. Due to the real-world context of the intervention, the number of antibiotic-related questions included in the midterm and final exams varied slightly between groups. These differences were related to the instructors’ autonomy in exam design, as well as variations in the integration of antibiotics into broader clinical case questions. For this reason, accuracy percentages were used instead of raw scores to allow meaningful comparison. In addition, intra-group comparisons (participants vs. non-participants within the same group) were conducted, where the assessment conditions were identical. Finally, the 11-question quiz, which was identical for all students who completed it, allowed us to assess the immediate impact of the Puzzle activity on knowledge consolidation in an homogeneous manner.

### Timeline of execution

All participants (intervention and control groups) completed the quiz within a two-week window in October 2024, shortly after the final theoretical session on antibiotics. This ensured comparable, though not identical, timing for the activity and/or questionnaires between groups. The midterm and final exams were conducted in November 2024 and January 2025, respectively.

### Satisfaction and perceived applicability

Student satisfaction and the perceived applicability of the activity were assessed through a simple ad hoc survey with two yes/no questions, about if they were satisfied with the activity and whether they found it helpful for consolidating their learning.

### Ethical considerations

Ethical approval was granted by the Ethics Committee of our institution (Code ID: CER052423), and the study was conducted in accordance with good research practice guidelines and current legislation. Informed consent was obtained from all participants. Participation was confidential, voluntary, and could be withdrawn at any time.

### Statistical analysis

The statistical analysis was conducted using SPSS v.30 (IBM Corp, Chicago, IL, USA). Results were expressed as percentages ± standard deviation (%±SD). Independent samples t-test was conducted to compare the accuracy percentages: (i) between the intervention groups vs. the control groups, and (ii) between the participants of Puzzle activity vs. the intra-group control students. One-way ANOVA was used to compare the three groups of intervention. When a significant effect was found, post hoc pairwise comparisons were performed using the Bonferroni correction to adjust for multiple comparisons. Chi-square test was performed to assess differences in categorical variables. Statistical significance was set at *p* < 0.05.

## Results

### Level of participation

A detailed breakdown of the total number of students (*n* = 330) and their participation percentages in each group is provided in Table 1, highlighting variability in engagement.

**Table 1:** Percentage of participation in the study. “Participants” refers only to students in intervention groups (A, B, and C) who took part in the Puzzle activity. “Intra-group controls” refers to students from the same intervention groups who did not participate. Control groups D and E followed only the standard theoretical course; students from all the groups presented completed the quiz and tests as part of scheduled assessment sessions

GROUP	PARTICIPANTS *N* (%)	INTRA-GROUP CONTROLS *N* (%)
PUZZLE INTERVENTION GROUPS		
A (*n* = 72)	39 (54.17)	33 (45.83)
B (*n* = 75)	20 (26.67)	55 (73.33)
C (*n* = 59)	24 (40.68)	35 (59.32)
Total Intervention	83 (40.29)	123 (59.70)
CONTROL GROUPS		
D (*n* = 71)	42 (59.15)	-
E (*n* = 53)	45 (84.91)	-
Total Control groups (*n* = 124)	87 (70.16)	-
Total (*n* = 330)	170 (51.51)	-

“Participants” refers only to students in intervention groups (A, B, and C) who took part in the Puzzle activity. “Intra-group controls” refers to students from the same intervention groups who did not participate. Control groups D and E followed only the standard theoretical course; the sample size in the second column correspond to students who completed the quiz as part of scheduled assessment sessions

When analyzing attendance at the Puzzle activity within the groups where the intervention was implemented, the overall participation level was calculated at 40.29%. Participation was higher in the group where the activity was integrated into the routine framework of a scheduled academic session, as outlined in the standard teaching plan (Group A, 54.17%). In contrast, Groups B and C, where the activity was conducted outside regular academic timeframes, showed significantly lower participation rates, with 26.67% and 40.68%, respectively (χ^2^ (1) = 16.07, p ± < 0.001).

Students attended the activity equipped with a diverse range of supportive materials. Observed pedagogical resources included notes, slides, printed documents, and infographics, which were either prepared in advance or accessed electronically during the in-person session.

### Results in the quiz questionnaire

**Fig. 2:**
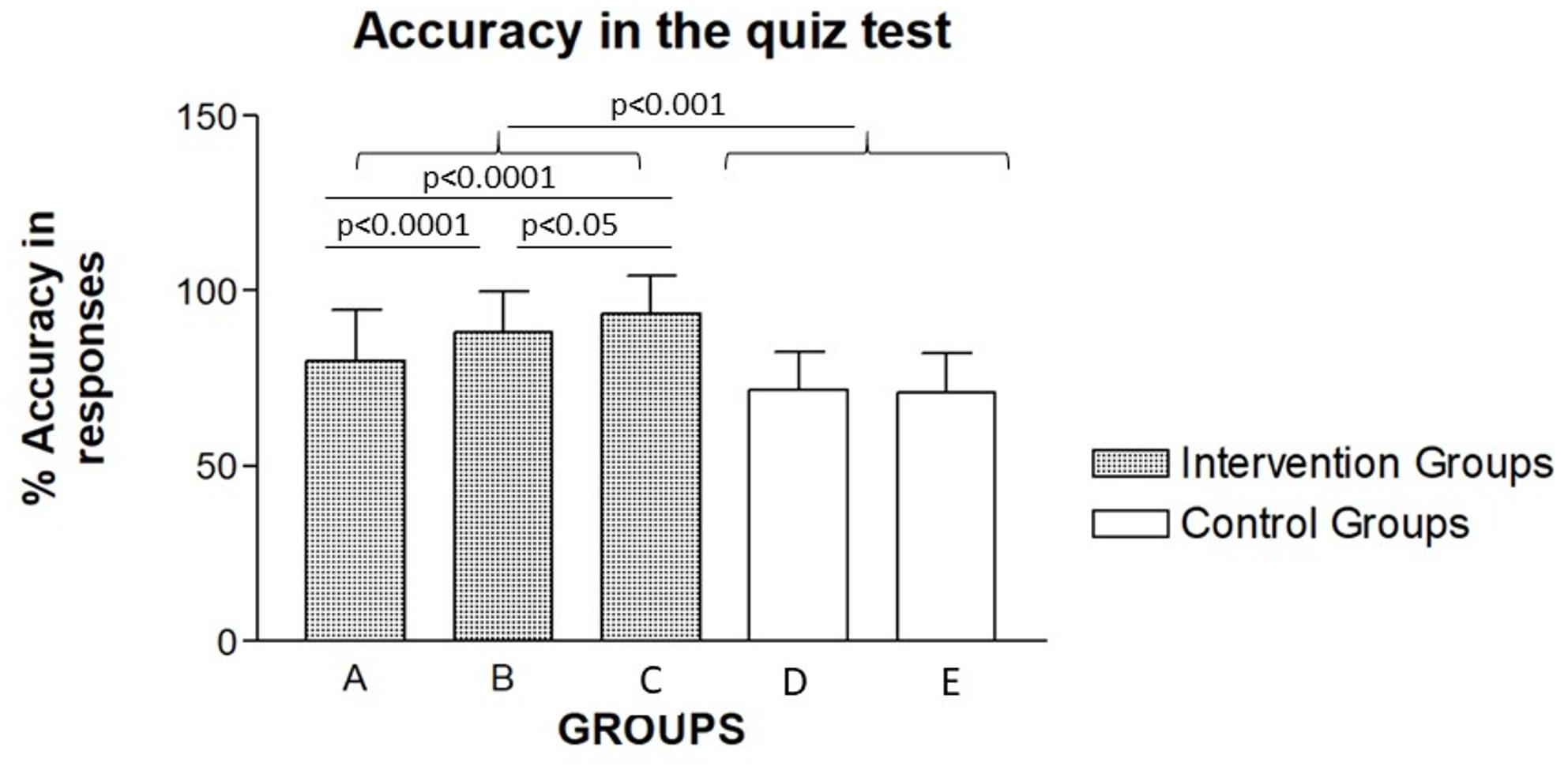
Accuracy percentages in the quiz questionnaire responses for all groups

Different percentages of accuracy were observed in the quiz administered at the end of the session (Fig. 2). Overall, the average accuracy among the intervention groups was calculated at 87.87 ± 11.39%, while the control groups displayed a lower average accuracy of 71.27% ± 11.24%. The results revealed a significant difference (t(32) = 4.01, *p* < 0.001), with the intervention groups showing significantly higher accuracy percentages compared to the control groups.

Among the intervention groups, Group A achieved an accuracy rate of 80.00% ± 14.69%, Group B reached 88.37 ± 11.39%, and Group C achieved the highest accuracy at 93.25 ± 10.88% in terms of correct answers. A one-way ANOVA was conducted to assess differences in accuracy rates among the three intervention groups (F = 25.89, *p* < 0.0001). Post hoc comparisons indicated that Group A performed significantly worse than both Group B (*p* < 0.0001) and Group C (*p* < 0.0001). Additionally, Group C demonstrated significantly higher accuracy compared to Group B (*p* < 0.05), suggesting graded accuracy values across the groups.

In contrast, among the control groups, Group D achieved 71.50 ± 11.24% accuracy, and Group E showed a similar performance with 71.04 ± 11.24% accuracy (p = NS) (Fig. 2).

### Results of the midterm exam

The number of correct and incorrect responses for each question about antibiotics included in this evaluative test, categorized by study group, is provided in Table 2. The total average accuracy percentage of the intervention groups was 80.61 ± 14.71% vs. 70.81 ± 3.04% in the control groups. The results revealed a significant difference in performance t(118) = 11.30, *p* < 0.001, with the intervention groups outperforming the control groups.

**Table 2:** Percentages of correct and incorrect responses for each antibiotic-related question formulated in the midterm exam

	GROUPS OF INTERVENTION						CONTROL GROUPS			
	GROUP A		GROUP B		GROUP C		GROUP D		GROUP E	
	CORRECT	INCORRECT	CORRECT	INCORRECT	CORRECT	INCORRECT	CORRECT	INCORRECT	CORRECT	INCORRECT
Question 1	79.17	20.83	68.00	32.00	98.31	1.69	87.32	12.68	90.38	9.62
Question 2	98.61	1.39	81.33	18.67	94.92	5.08	63.38	36.62	69.23	30.77
Question 3	94.44	5.56	61.33	38.67	74.58	25.42	95.77	4.23	61.54	38.46
Question 4	97.22	2.78	98.67	1.33	96.61	3.39	64.79	35.21	96.15	3.85
Question 5	81.94	18.06	72.00	28.00	93.22	6.78	53.52	46.48	82.69	17.31
Question 6	61.11	38.89	90.67	9.33	89.83	10.17	-	-	38.46	61.54
Question 7	73.61	26.39	46.67	53.33	88.14	11.86	-	-	82.69	17.31
Question 8	94.44	5.56	16.00	84.00	88.14	11.86	-	-	32.69	67.31
Question 9	94.44	5.56	57.33	42.67	79.66	20.34	-	-	78.85	21.15
Question 10	91.67	8.33	88.00	12.00	83.05	16.95	-	-	53.85	46.15
Question 11	-	-	48.00	52.00	83.05	16.95	-	-	92.31	7.69
Question 12	-	-	-	-	100.00	0.00	-	-	48.08	51.92
Question 13	-	-	-	-	91.53	8.47	-	-	73.08	26.92
Question 14	-	-	-	-	-	-	-	-	73.08	26.92
AVERAGE	86.67	13.33	68.00	32.00	88.64	11.36	72.96	27.04	68.65	31.35
SD	12.30	12.30	24.42	24.42	7.67	7.67	40.24	18.53	21.61	21.61

“Question 1”, “Question 2”, etc., serve as numerical references for each row and do not correspond to the same item across all groups. Due to curricular autonomy, the content and number of antibiotic-related exam questions varied slightly between teaching groups. “–” indicates that the corresponding question was not administered to that group, due to differences in exam content. This variation reflects the academic autonomy of instructors in designing assessments

Average percentages of accuracy in the responses of the intervention groups in the midterm exam were 86.67 ± 12.30% (Group A), 68.00 ± 24.42% (Group B) and 88.64 ± 7.67% (Group C), whereas average percentages in the control groups were 72.96 ± 40.24% (Group D) and 68.65 ± 21.61% (Group E). Differences in accuracy rates among the three intervention groups were observed (F = 4.10, *p* < 0.05). Post hoc comparisons indicated that Group B and C performed differently (*p* < 0.05), while no differences were found among control groups (p = NS).

**Fig. 3:**
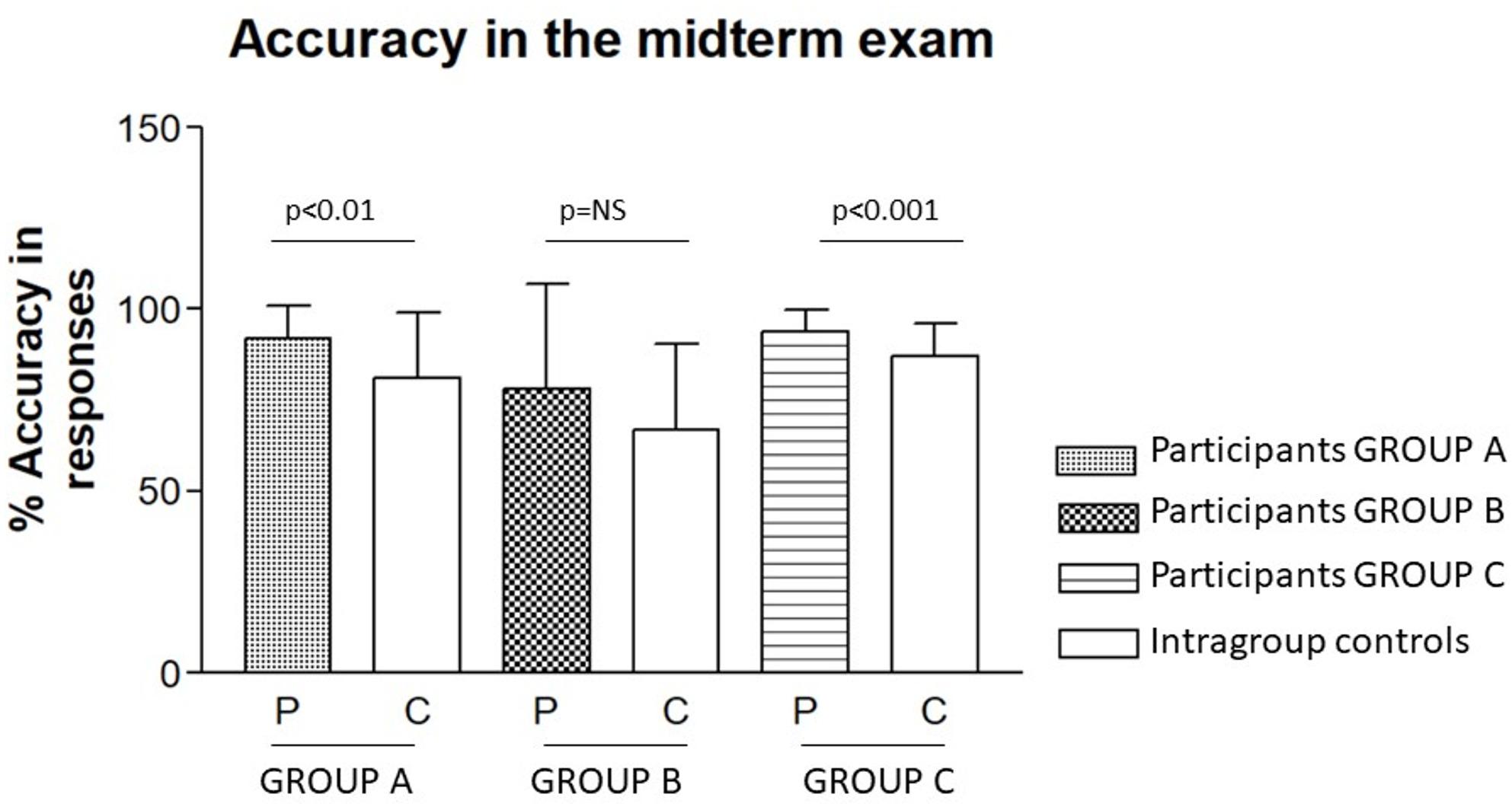
Percentages of correct responses of participants and intra-group controls for each antibiotic-related question formulated in the midterm exam, in the intervention groups. P, participants; C, controls. Accuracy percentages in antibiotic-related questions from the midterm exam, comparing students who participated in the Puzzle activity with their intra-group peers who did not. These within-group comparisons (Groups A, B, and C) were conducted to control for contextual factors such as teaching staff and exam content

Accuracy outcomes of participants and intra-group controls of each intevention group are shown (Fig. 3). Participants of Group A answered with an accuracy of 92.11 ± 8.68%, whereas non-participating students achieved an accuracy of 80.88 ± 18.20% (t(58) = 3.05, *p* < 0.01). In Group B, participants exhibited an accuracy of 78.13% ± 16.67, compared to their intra-group controls, who scored 66.78 ± 23.95%, (t(58) = 0.90, p = NS). Finally, in Group C, students who participated in the activity responded with an accuracy of 93.68 ± 6.93%, while their non-participating peers achieved 86.25 ± 8.99, (t(58) = 3.67, *p* < 0.001). Percentages of correct responses of participants and intra-group controls for each antibiotic-related question formulated in the midterm exam in the intervention groups are provided in Supplementary Material (Table S3).

### Results of the final exam

The number of correct and incorrect responses for each question about antibiotics included in this evaluative test, categorized by study group, is provided in Table 3. The total average accuracy percentage of the Puzzle groups was 75.16 ± 12.21% vs. 48.14 ± 13.30% in the control groups. The results revealed a significant difference in performance t(118) = 15.52, *p* < 0.001, with the intervention groups significantly outperforming the control groups.

**Table 3:** Percentages of correct and incorrect responses for each antibiotic-related question formulated in the final exam

QUESTIONS	GROUPS OF INTERVENTION						CONTROL GROUPS			
	GROUP A		GROUP B		GROUP C		GROUP D		GROUP E	
Question 1	84.29	15.71	61.33	38.67	55.93	44.07	39.44	60.56	77.36	22.64
Question 2	94.29	5.71	69.33	30.67	72.88	27.12	38.03	61.97	39.62	60.38
Question 3	85.71	14.29	100.00	0.00	66.10	33.90	-	-	79.25	20.75
Question 4	74.29	25.71	66.67	33.33	44.07	55.93	-	-	33.96	66.04
Question 5	-	-	92.00	8.00	74.58	25.42	-	-	84.91	15.09
Question 6	-	-	-	-	64.41	35.59	-	-	-	-
AVERAGE	84.64	15.36	74.33	25.67	59.75	40.25	38.73	61.27	57.55	42.45
SD	8.20	8.20	17.43	17.43	12.56	12.56	1.00	1.00	24.09	24.09

“Question 1”, “Question 2”, etc., serve as numerical references for each row and do not correspond to the same item across all groups. Due to curricular autonomy, the content and number of antibiotic-related exam questions varied slightly between teaching groups. “–” indicates that the corresponding question was not administered to that group, due to differences in exam content. This variation reflects the academic autonomy of instructors in designing assessments

Average percentages of accuracy in the responses of the intervention groups in the final exam were 84.64 ± 8.20% (Group A), 74.33 ± 17.43% (Group B) and 59.75 ± 12.56% (Group C), whereas average percentages in the control groups were 38.73 ± 1.00% (Group D) and 57.55 ± 24.09% (Group E). Accuracy rates among the three intervention groups and the two control groups, respectively, did not lead to significant differences (F = 1.37, p = NS).

**Fig. 4:**
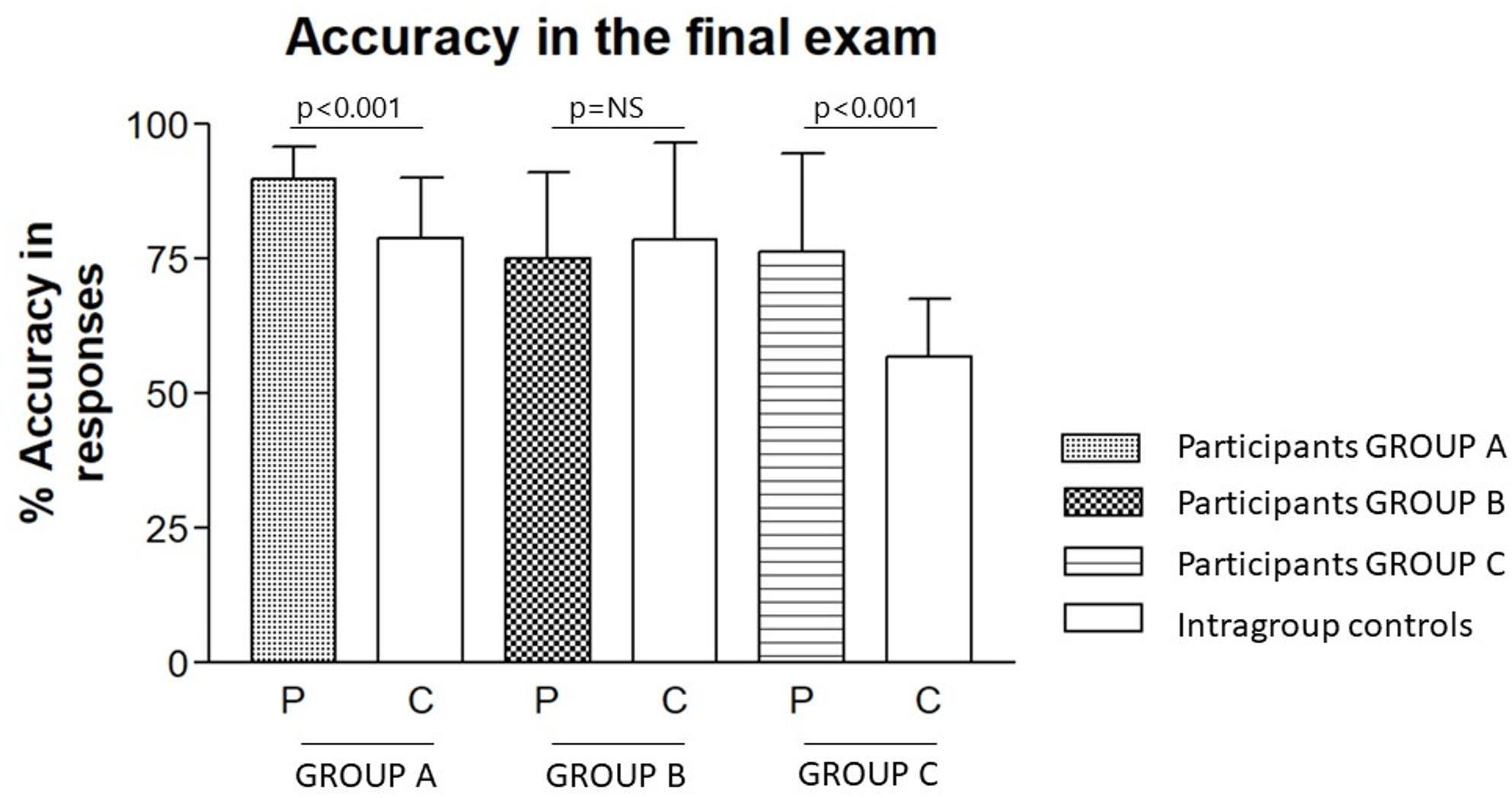
Percentages of correct responses of participants and intra-group controls for each antibiotic-related question formulated in the final exam, in the intervention groups. Accuracy percentages in antibiotic-related questions from the final exam, comparing students who participated in the Puzzle activity with their intra-group peers who did not. These comparisons reflect performance differences within the same intervention groups, under identical teaching and testing conditions

Accuracy outcomes of participants and intra-group controls of each intervention group are shown (Fig. 4). Independent t-tests comparing final exam accuracy between intra-group participants and non-participants revealed significant differences in two out of the three groups. Specifically, regarding Group A, participants answered with an accuracy of 89.86 ± 46.64, whereas non-participating students achieved an accuracy of 78.79 ± 41.62%, (t(58) = 4.73, *p* < 0.001). In Group B participants exhibited an accuracy of 75.00 ± 33.77%, compared to their intra-group controls, who scored 78.64 ± 35.84%, (t(58) = − 0.83, p = NS). Finally, in Group C, students who participated in the activity responded with an accuracy of 76.32 ± 18.16%, while their non-participating peers achieved 56.67 ± 10.92%, (t(58) = 5.08, *p* < 0.001). Percentages of correct responses of participants and intra-group controls for each antibiotic-related question formulated in the final exam in the intervention groups are provided in Supplementary Material (Table S4).

### Results on satisfaction levels and perceived applicability

**Fig. 5:**
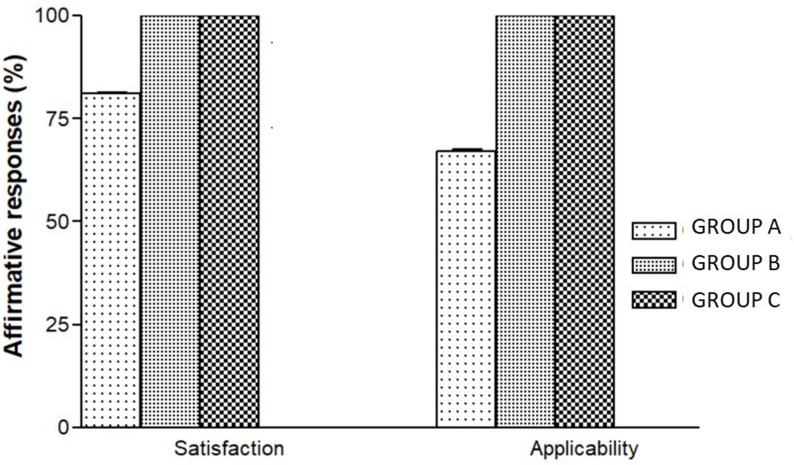
Percentages of reported satisfaction with the activity and perceived applicability of the activity in the intervention groups

The students who participated in the intervention expressed high levels of satisfaction and perceived usefulness of the activity (Fig. 5). In Group A, 81% of the students reported satisfaction with the activity. Meanwhile, in Groups B and C, 100% of the students reported satisfaction with the activity.

Regarding applicability, when asked if the activity was perceived as useful for consolidating their learning (applicability), 67% of students in Group A responded affirmatively. Similarly, in Groups B and C, the 100% of the students answered positively, highlighting the perceived value of the intervention.

### Survey perception of satisfaction and applicability in learning consolidation

## Discussion

Two significant challenges from different paradigms led us to shape the objective of this study: in the academic setting, the issue of university student demotivation, and in the clinical setting, the global rise in bacterial resistance along with the increasing role of nurses in prescribing medications. Given these challenges, our focus was on enhancing nurse training in the pharmacological field, particularly regarding antibiotics. Both challenges converge in the urgent need for enhanced educational programs targeting antimicrobial knowledge and rational use within the healthcare context, as previously acknowledged [19]. Indeed, this is not the first time that issues in the training of healthcare personnel have been identified [20], nor the first attempt to work on implementing improvements in this regard [21]. The Puzzle technique was chosen not only to support memorization-intensive content but also to foster active engagement and peer-to-peer knowledge exchange. Our findings show that the Puzzle pedagogical activity improved learning among nursing students. Intervention groups achieved significantly higher quiz and exam accuracy percentages than controls. Additionally, participants outperformed their intra-group controls, reinforcing the effectiveness of this approach.

Participation rates varied across groups, with Group A showing lower engagement compared to Groups B and C, which may have influenced how effectively students benefited from the intervention. In our experience, the integration of the activity within the regular academic schedule resulted, as expected, in higher participation rates compared to the groups where the activity was conducted during a complementary session specifically designed for this purpose, probably due to the additional time dedication of the students and the extra efforts to adjust the course schedule and personal calendar. Accordingly, the literature already reports significantly higher participation in mandatory activities compared to voluntary ones [22].

Students participated in the activity carrying a variety of supplementary resources, reflecting the students’ resourcefulness and adaptability in engaging with the activity. The instructors made a deliberate choice not to impose any specific requirements regarding the materials students needed to bring. This approach aimed to maintain neutrality and avoid influencing the selection of resources or restricting students’ autonomy. Although this decision introduced a potential risk of disparity and perceived unfairness due to the varying quality of materials freely contributed by students, there was no evidence of dissatisfaction or perceived injustice reported by the participants at any point.

The post-intervention quiz results suggest that the Puzzle activity effectively supported short-term learning consolidation, particularly regarding the classification and clinical use of antibiotics. Similarly, response accuracy in both midterm and final exams was consistently higher among students who participated in the intervention, indicating potential medium- and long-term benefits. Intra-group comparisons further reinforce this pattern, especially in groups A and C. However, the absence of significant differences in group B may be related to lower participation rates and greater variability in accuracy percentages, limiting the strength of conclusions in that subgroup. These findings can be interpreted through several educational theories. First, the Puzzle technique aligns with constructivist principles [23], as students actively build knowledge through collaboration and peer teaching. Second, by dividing complex pharmacological content into smaller, manageable segments, the activity helps to reduce cognitive load [24], enhancing comprehension and retention. Finally, the intervention may also engage key dimensions of the Self-Determination Theory [25], by fostering a sense of autonomy, competence, and social connection, all of which are known to enhance intrinsic motivation. Together, these frameworks support the pedagogical value of the Puzzle approach in demanding academic contexts.

Previous results showed effectiveness of this same pedagogic technique in the nursing context, and reported improved psychomotor skill levels and increased retention of knowledge in nursing first year students [26]. Similar positive experiences have also been described in the literature among Bachelor of Medicine, and Bachelor of Surgery students, among which cooperative learning like Puzzle facilitated learning allowing student-student discussion, improving communication and teaching skills [27]. An increasing body of evidence points to the effectiveness of this peer-to-peer activity in the nursing and medical context, not only in aiding memoristic learning as exemplified herein but also in ethic and legal fields concerning malpractice or medical errors in nursing practice, among others [28]. Furthermore, the level of satisfaction reported by the students encourages us to propose new editions of this initiative, incorporating enhancements which may foster greater student participation. Taken together, our findings derived from the quiz, the midterm and the final exams, suggested that, in general, the implementation of this intervention had a positive impact. Nevertheless, the role of nursing educators as content experts remains essential to guide, validate, and consolidate knowledge, ensuring that peer-to-peer learning complements rather than replaces faculty-led instruction.

This study is not exempt from limitations, including the heterogeneity of the questions posed across different tests, the materials used by students, and the timing of the evaluative tests, among other factors. Although the quiz was administered within a coordinated limited time window, we cannot fully exclude the potential influence of minor variations in timing across groups. Despite the temporal proximity of one to two weeks between groups, the scheduling of ad hoc sessions outside the standard timetable may still have influenced students’ participation and performance. Another notable limitation of this study is the potential for self-selection bias, as participation in the Puzzle activity was voluntary. It is possible that students who chose to participate were generally more engaged and intrinsically motivated, factors that could have contributed to their higher performance. In fact, previous studies have reported that students with a history of high performance tended to participate more in flipped classroom sessions compared to those with lower performance [29]. As a result, the observed differences in our outcomes may not be solely attributable to the activity itself but also to pre-existing motivational differences between participants and non-participants. The unequal distribution of three intervention groups and two control groups was due to logistical factors, which may represent a limitation. Although demographic data were not collected at the individual level, institutional records show a relatively homogeneous cohort (predominantly female, 19–22 years), which reduces intra-group differences but still limits generalizability. Another limitation of the study lies in the variability in the number of antibiotic-related questions across exam groups. This heterogeneity arose from the real-world academic setting, where instructors independently designed their assessments. Although efforts were made to standardize the antibiotic content coverage, achieving complete uniformity across all groups was not feasible. To address this issue, we analyzed accuracy percentages rather than absolute scores and prioritized intra-group comparisons, providing more reliable insights into the impact of the intervention. Another limitation is that information on students’ prior academic performance (e.g., grade point averages or equivalent records) was not available, and therefore group allocation could not be adjusted for baseline academic strength. Finally, the satisfaction survey included only yes/no questions, which restricted the depth of qualitative feedback, therefore, no further insights could be obtained to explain why a subset of students reported lower satisfaction. Future implementations may consider using Likert scales and open-ended items to gain more detailed insights into students’ perceptions and experiences. We cannot exclude the possibility that students felt subtle pressure to provide favorable responses. Although no pilot test of the activity or quiz was conducted in this initial implementation, future applications may benefit from a small-scale pre-test to identify and address potential issues related to clarity, timing, or usability before broader deployment. Globally, variability in exam questions and in the resources used may limit the standardization and reproducibility of our findings in other contexts; however, we believe it is still valuable and useful to share this pilot experience with the academic community.

The differences observed between groups could also be attributed to variations in teaching styles among instructors, which represent a variable factor that may interfere with direct group comparisons. The lower participation rate in group B may be partially explained by the fact that this group conducted the Puzzle activity during an afternoon session. Differences in scheduling and individual availability could have contributed to the reduced engagement in this particular cohort. However, this does not diminish the relevance of the findings, as the use of intra-group controls bypasses this variable in the analyses conducted within the intervention groups. Additionally, in two out of the three intervention groups, participants demonstrated higher response accuracy, further supporting the positive impact of the activity. Despite these constraints, sharing experiences like this with the broader healthcare community, including both clinicians and educators, is crucial for fostering improvements in education quality, particularly when supported by positive outcomes and well-received student feedback, as demonstrated in this study.

The novelty of this study lies not in the pedagogical technique itself, but in its application to antibiotic pharmacology within undergraduate nursing education, using a multi-cohort design and both intra-group and external control comparisons.

It is important to recognize that the nurses in the classroom today will become the future bedside nurses in hospitals, and it is essential to promote excellence in their training to ensure the highest quality of life for future patients [30]. In conclusion, our findings support the value of innovative peer-to-peer strategies such as the Puzzle technique as a complementary learning modality in Clinical Pharmacology, with potential to enhance students’ comprehension of complex content. Studies like this one, which share pedagogical experiences, can assist the educational community in improving both the functioning and learning processes among students, something not negligible, considering the potential impact this may later have on society in clinical settings. Indeed, it has been observed that nurse students harbor fears related to the clinical environment that influence their participation and learning [31] which highlights the need to join efforts in minimizing these insecurities within this sector.

## Conclusions

Our findings specifically pertain to undergraduate nursing students and undergraduate nursing education. The implementation of the Puzzle technique in the Clinical Pharmacology course significantly improved nursing students’ accuracy in antibiotic-related content and appeared to foster engagement, as suggested by participation trends and satisfaction levels. Students who participated in the activity obtained higher accuracy percentages in both midterm and final assessments compared to their peers, suggesting a positive effect of the intervention on short, medium, and long-term knowledge retention. Additionally, students reported high levels of satisfaction and perceived applicability of the activity. These findings support the integration of peer-to-peer collaborative methodologies as effective pedagogical tools in nursing education, particularly for content-heavy and memorization-intensive subjects. Future implementations could explore strategies to enhance participation rates and assess the long-term impact on clinical decision-making skills.

## Supplementary Information

Supplementary Material 1.

## Data Availability

Most of the data generated and analysed during this study are included in this published article and its supplementary information files. Additional details are available from the corresponding author upon reasonable request.
