# Supplementary material for: Puzzle-based pedagogic activity to address rote learning of antibiotic classes in clinical pharmacology course for undergraduate nursing students
Source: BMC Med Educ. 2026 May 28;26:1209. doi: 10.1186/s12909-026-09484-1 (PMC13404095; doi:10.1186/s12909-026-09484-1)
Supplement: Supplementary file 1 — Supplementary Material 1. [file 12909_2026_9484_MOESM1_ESM.docx]

**SUPPLEMENTARY MATERIAL S1**

**Quiz Questions**

**Which antibiotic can we prescribe to a pregnant patient?**

- Penicillins
- Quinolones
- Aminoglycosides
- Macrolides

**Penicillins are natural:**

- Yes. because they derive from the mold of the Penicillium fungus
- Not all them: there are also synthetic forms like carboxypenicillins or aminopenicillins

**Streptogramins, chloramphenicol, lincosamides, linezolid, and macrolides:**

- Are inhibitors of bacterial protein synthesis
- Alter the bacterial plasma membrane
- Attack bacterial DNA

**For a patient allergic to penicillin. I can administer as an alternative:**

- Benzylpenicillin
- Oxacillin or dicloxacillin
- I have to suspend antibiotic treatment
- Cephalosporins

**Vancomycin is associated with:**

- Red man syndrome
- Gray man syndrome
- Its administration is not associated with adverse reactions

**Tetracyclines:**

- Are inhibitors of the cell wall
- Do not cause dental or bone-related adverse reactions
- Are not administered to neonates or pediatric patients

**What can we cause to a developing fetus if we administer aminoglycosides?**

- Nothing. it is safe for the fetus
- Irreversible deafness due to its ototoxicity
- Teratogenic effects
- Abortion and malformations

**Bacteriostatic means:**

- It inhibits bacterial proliferation
- It kills and eliminates bacteria

**Fosfomycin is recommended for:**

- Urinary tract infections (UTIs)
- Inner ear infections

**SUPPLEMENTARY MATERIAL S2**

**Exam questions**

**A bacteriostatic drug means:**

1. That the antimicrobial destroys the germ
2. That the antimicrobial has a broad antimicrobial action. that is. it attacks various bacteria. fungi or viruses
3. That the antimicrobial temporarily inhibits the growth or multiplication of the germ
4. All the above are false

**Choose the correct answer regarding antibacterial mechanisms of action. Select the one related to the beta-lactam antibiotic group.**

1. Inhibition of bacterial cell wall synthesis
2. Disruption of the cytoplasmic membrane
3. Inhibition of protein synthesis
4. Interference with the synthesis and/or metabolism of nucleic acids

**Choose the correct answer. Which penicillin is administered orally?**

1. Penicillin G sodium
2. Penicillin G benzathine
3. Ticarcillin
4. Amoxicillin

**Choose the correct answer. Which penicillin is called anti-staphylococcal because it is resistant to beta-lactamase?**

1. Amoxicillin
2. Penicillin G
3. Ticarcillin
4. Cloxacillin

**Which of the following effects is a potential complication of excessive or incorrect use of antibiotics?**

1. High blood pressure
2. Type 1 diabetes
3. Bacterial resistance
4. Kidney failure

**Which of the following is NOT an example of an antibiotic?**

1. Ampicillin
2. Diclofenac
3. Ceftriaxone
4. Azithromycin

**What type of infections are carbapenems like imipenem most likely to treat?**

1. Respiratory infections
2. Urinary tract infections
3. Infections caused by resistant bacteria
4. Viral infections

**Which of the following antibiotics belongs to the penicillin family?**

1. Cephalexin
2. Meropenem
3. Amoxicillin
4. Ciprofloxacin

**Which of the following is a sign of an allergic reaction to an antibiotic?**

1. Itching and hives
2. Dry mouth
3. Excessive thirst
4. Blurred vision

**Which of the following antibiotics is commonly used to treat urinary tract infections?**

1. Metronidazole
2. Ceftriaxone
3. Fosfomycin
4. Erythromycin

**Which of the following antibiotics is contraindicated in pregnant women due to its effect on fetal development?**

1. Cephalosporins
2. Penicillin
3. Aminoglycosides
4. Azithromycin

**Which type of antibiotic can be bacteriostatic or bactericidal depending on its dose?**

1. Beta-lactams
2. Nystatin
3. Tetracyclines
4. Quinolones

**Penicillin G + procaine and penicillin G benzathine:**

1. Are used as the first choice for dental infections
2. Are administered intramuscularly
3. Are indicated when low but prolonged doses are desired
4. Both b and c are correct

**Streptogramins, chloramphenicol, lincosamides, linezolid and macrolides are:**

1. Glycopeptides and lipopeptides
2. Inhibitors of bacterial protein synthesis
3. Interfering with nucleic acid metabolism
4. Inhibitors of folic acid pathway

**Which of these statements is correct?**

1. There are several generations of cephalosporins
2. Beta-lactam antibiotics have a narrow spectrum
3. Imipenem can be administered via rapid infusion
4. None of the above is correct

**During antibiotic administration:**

1. We will prioritize using the smallest gauge
2. We will avoid mixing aminoglycosides with beta-lactams in the same vial
3. We will use the same injection site for the different intramuscular administrations
4. We will prepare the dilution or mixture as far in advance as possible before administration

**SUPPLEMENTARY MATERIAL TABLE S3**

**Supplementary Material Table S3.** Percentages of correct responses of participants and intra-group controls for each antibiotic-related question formulated in the midterm exam, in the intervention groups.

|  | **INTERVENTION GROUPS** | | | | | |
| --- | --- | --- | --- | --- | --- | --- |
|  | **GROUP A** | | **GROUP B** | | **GROUP C** | |
|  | **INTERVENTION** | **CONTROLS** | **INTERVENTION** | **CONTROLS** | **INTERVENTION** | **CONTROLS** |
| Question 1 | 86.84 | 67.65 | 68.75 | 67.80 | 100.00 | 97.5 |
| Question 2 | 100.00 | 97.06 | 87.50 | 79.66 | 94.74 | 95 |
| Question 3 | 97.37 | 91.18 | 87.50 | 54.24 | 78.95 | 72.50 |
| Question 4 | 100.00 | 97.06 | 100.00 | 98.31 | 100.00 | 95.00 |
| Question 5 | 84.21 | 79.41 | 56.25 | 76.27 | 89.47 | 95.00 |
| Question 6 | 73.68 | 44.12 | 100.00 | 88.14 | 100.00 | 85.00 |
| Question 7 | 89.47 | 58.82 | 62.50 | 42.37 | 100.00 | 82.50 |
| Question 8 | 97.37 | 94.12 | 68.75 | 20.34 | 94.74 | 85.00 |
| Question 9 | 100.00 | 88.24 | 93.75 | 54.24 | 89.47 | 75.00 |
| Question 10 | 92.11 | 91.18 | 56.25 | 86.44 | 89.47 | 80.00 |
| Question 11 | - | - | - | 45.76 | 94.74 | 77.50 |
| Question 12 | - | - | - | - | 100.00 | 100 |
| Question 13 | - | - | - | - | 94.74 | 90 |
| **AVERAGE** | 92.11 | 80.88 | 78.13 | 66.78 | 93.68 | 86.25 |
| **SD** | 8.68 | 18.20 | 16.67 | 23.95 | 6.93 | 8.99 |

**SUPPLEMENTARY MATERIAL TABLE S4**

**Supplementary Material Table S4.** Percentages of correct responses of participants and intra-group controls for each antibiotic-related question formulated in the final exam in the intervention groups.

|  | **INTERVENTION GROUPS** | | | | | |
| --- | --- | --- | --- | --- | --- | --- |
|  | **GROUP A** | | **GROUP B** | | **GROUP C** | |
|  | **INTERVENTION** | **CONTROLS** | **INTERVENTION** | **CONTROLS** | **INTERVENTION** | **CONTROLS** |
| Question 1 | 91.89 | 75.76 | 68.75 | 59.32 | 73.68 | 47.50 |
| Question 2 | 94.59 | 93.94 | 62.50 | 71.19 | 78.95 | 70.00 |
| Question 3 | 91.89 | 78.79 | 100.00 | 100.00 | 94.74 | 52.50 |
| Question 4 | 81.08 | 66.67 | 62.50 | 67.80 | 42.11 | 45.00 |
| Question 5 | - | - | 81.25 | 94.92 | 84.21 | 70.00 |
| Question 6 | - | - | - | - | 84.21 | 55.00 |
| **AVERAGE** | 89.86 | 78.79 | 75.00 | 78.64 | 76.32 | 56.67 |
| **SD** | 46.64 | 41.62 | 33.77 | 35.84 | 18.16 | 10.92 |

**
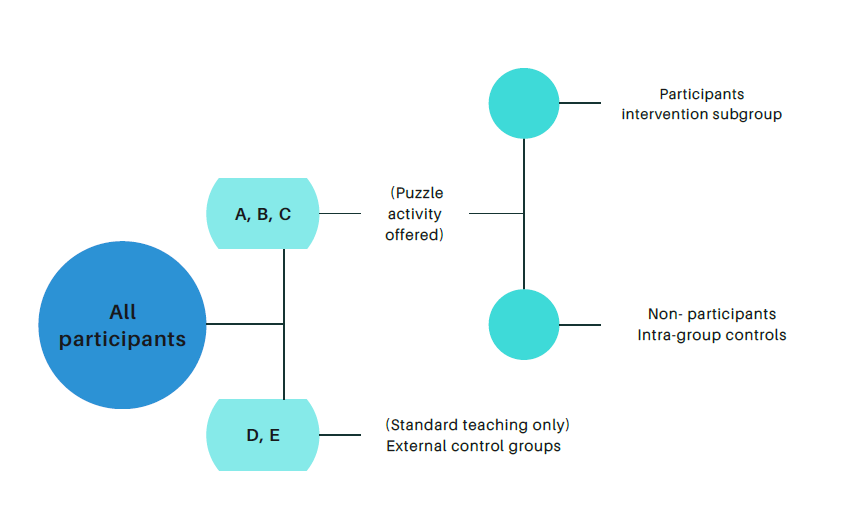
SUPPLEMENTARY MATERIAL FIGURE S1**

**Supplementary Figure 1.** Overview of group allocation and comparison strategy. Groups A to C correspond to cohorts in which the Puzzle activity was offered, including participants and intra-group controls, while groups D and E received standard teaching only.
